# Supplementary figures and images for: A multi-population-based genomic analysis uncovers unique haplotype variants and crucial mutant genes in SARS-CoV-2
Source: J Genet Eng Biotechnol. 2022 Nov 1;20:149. doi: 10.1186/s43141-022-00431-3 (PMC9626712; doi:10.1186/s43141-022-00431-3)

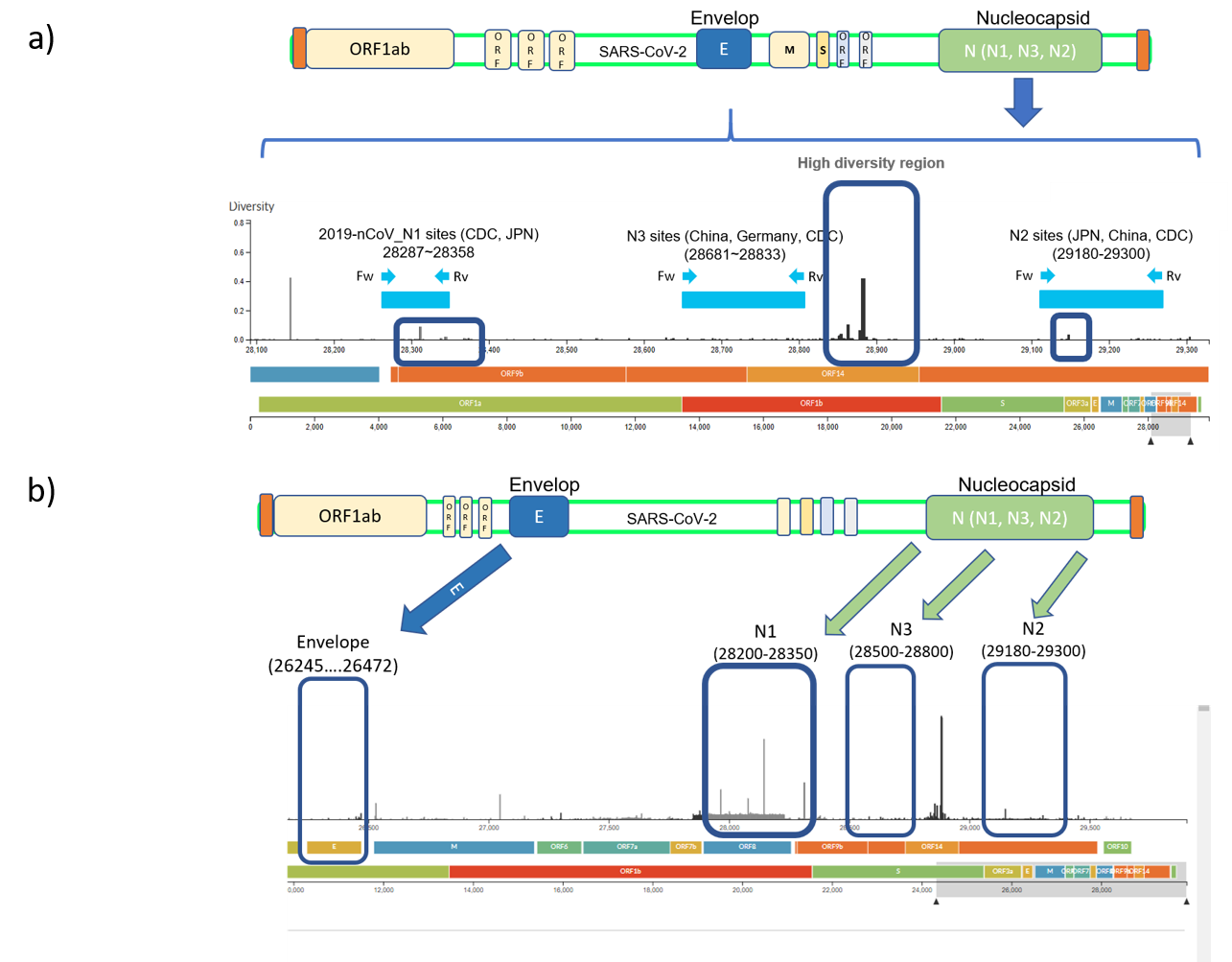

Supplement: Supplementary file 1 — Additional file 1: Supplementary Figure 1. Structural representation of SARS-CoV-2 and primer/probe sites. a) global target detection (primer/probe binding) sites and b) representation envelop and nucleocapsid region. The diversity sites were sourced from Hadfield et al. (2018). [file 43141_2022_431_MOESM1_ESM.tif]
